# Supplementary material for: Relative income and its relationship with mental health in UK employees: A systematic review
Source: PLoS One. 2025 Apr 23;20(4):e0320402. doi: 10.1371/journal.pone.0320402 (PMC12017527; doi:10.1371/journal.pone.0320402)
Supplement: Attachment — Submitted filename: Response to Reviewers.docx [file pone.0320402.s006.docx]

Dear Editor and Reviewer,

Thank you for taking the time to review our manuscript. I have detailed below how I have responded to the reviewer comments as well as any updates that are required to financial disclosure statements.

**Journal requirements**

- We have now updated the manuscript to meet PLOS ONE’s style requirements and made the other requested changes.
- The reference list has been checked for accuracy, and a full proofread has been conducted.
- We have updated the manuscript's funding information section and the submission system.
- For the competing interests section, we have now added in a line to confirm: This does not alter our adherence to PLOS ONE policies on sharing data and materials. Please update the online submission form on our behalf.

**Response to reviewer comments**

| **Reviewer point** | **Response** | **Location of change** |
| --- | --- | --- |
| For introduction, including relevant statistics, such as prevalence rates and correlation coefficients of relative income and mental health would strengthen the argument and provide a more robust foundation for the claims made. | Thank you for your comment. We have added in further evidence regarding relative income effects and included statistical information. As many used ordered probit regression, the coefficients are not easily interpretably on their own so we have presented the statistical evidence as percentage changes or point changes. | Introduction: lines 98-103 |
| For method, please provide the full form of the term 'PRISMA guidelines' and 'PROSPERO' when first mentioned in the text to ensure clarity for readers who may not be familiar with this abbreviation. | Thank you for raising this. I have now spelled out these abbreviations in full. | Methods: lines 151-154 |
| Next, include the complete keywords and query strings or search strategies used for all nine (9) electronic databases and provide them as an appendix to enhance transparency and allow readers to assess the comprehensiveness and reproducibility of the literature search. | Thank you for your comment. The full strategy including all the terms used for all nine databases is already included in supplementary material 2. There were approximately ten terms search terms for each concept and therefore it is not feasible to include all of these in the main text. | N/A |
| Table 1 and Table 2 can be made more compact by minimising the spacing between rows and columns. This will improve readability and reduce unnecessary white space. | Thank you for your comment. These tables have now been amended. | Table 1 and Table 2 |
| For results, at least a reference for the Quality Assessment method must be provided and include a thorough explanation of the scoring calculation process to clearly show how the quality of studies was evaluated and scored. | Thank you for your comment. The Quality Assessment method has been cited in the methods section. We have added more information into the methods section to show how the quality of studies was evaluated and scored. In addition, we have given further description in the results section of the criteria for a poor rating. | Methods: lines 214-222  Results: lines 298-304 |
| For discussion, ensure that the discussion section goes beyond merely stating the findings. Provide a thorough analysis by crystallising the key insights, drawing connections between the results and explaining their implications in the context of existing literature. Additionally, elaborate on how the findings could be applied in practice to address poverty and health issue. | Thank you for your comment. We have restructured the discussion and added in further insights. In particular, we have expanded the previous literature section and elaborated on how the findings can be applied to address poverty. | Discussion: whole section |
