# Supplementary material for: Relative income and its relationship with mental health in UK employees: A systematic review
Source: PLoS One. 2025 Apr 23;20(4):e0320402. doi: 10.1371/journal.pone.0320402 (PMC12017527; doi:10.1371/journal.pone.0320402)
Supplement: Attachment — Submitted filename: Response to Reviewers_RelativeIncomeReview.docx [file pone.0320402.s007.docx]

Dear reviewer,

Thank you for reviewing our paper and for your feedback. Please see below our responses to your comments.

1. **Figure 3 should include wellbeing as part of the outcome.**

*Thank you for your comment. We have now included this in figure 3. We also realised these should not be part of the main manuscript so please see separate figure files.*

**2. An explanation of moderation should be added to help readers better understand the effect of these moderating factors.**

*Thank you for your comment. We have added in an explanation of moderating factors on line 287-289. The specific effect of all moderators is already included under the relevant sub-headings (e.g. income inequality).*

Yours Sincerely,

Bethany Croak on behalf of all authors
